# Positron Emission Tomography reveals age-associated hypothalamic microglial activation in women

**DOI:** 10.1038/s41598-022-17315-8

**Published:** 2022-08-03

**Authors:** Tracy Butler, Lidia Glodzik, Xiuyuan Hugh Wang, Ke Xi, Yi Li, Hong Pan, Liangdong Zhou, Gloria Chia-Yi Chiang, Simon Morim, Nimmi Wickramasuriya, Emily Tanzi, Thomas Maloney, Patrick Harvey, Xiangling Mao, Qolamreza Ray Razlighi, Henry Rusinek, Dikoma C. Shungu, Mony de Leon, Craig S. Atwood, P. David Mozley

**Affiliations:** 1 Department of Radiology, Weill Cornell Medicine, New York, USA; 2 Department of Psychiatry, Brigham and Women’s Hospital, Boston, USA; 3 Department of Radiology, New York University, New York, USA; 4 Department of Gerontology, University of Wisconsin, Madison, Madison USA; 5 Department of Radiology, Brain Health Imaging Institute, Weill Cornell Medicine, 405 E 61st St, New York, NY 10065 USA

**Keywords:** Neural ageing, Magnetic resonance imaging, Molecular imaging, Positron-emission tomography, Neurodegeneration, Neurology, Alzheimer's disease, Microglia, Neuroscience, Sexual dimorphism, Neuroendocrine diseases, Ageing, Menopause, Brain

## Abstract

In rodents, hypothalamic inflammation plays a critical role in aging and age-related diseases. Hypothalamic inflammation has not previously been assessed in vivo in humans. We used Positron Emission Tomography (PET) with a radiotracer sensitive to the translocator protein (TSPO) expressed by activated microglia, to assess correlations between age and regional brain TSPO in a group of healthy subjects (n = 43, 19 female, aged 23–78), focusing on hypothalamus. We found robust age-correlated TSPO expression in thalamus but not hypothalamus in the combined group of women and men. This pattern differs from what has been described in rodents. Prominent age-correlated TSPO expression in thalamus in humans, but in hypothalamus in rodents, could reflect evolutionary changes in size and function of thalamus versus hypothalamus, and may be relevant to the appropriateness of using rodents to model human aging. When examining TSPO PET results in women and men separately, we found that only women showed age-correlated hypothalamic TSPO expression. We suggest this novel result is relevant to understanding a stark sex difference in human aging: that only women undergo loss of fertility—menopause—at mid-life. Our finding of age-correlated hypothalamic inflammation in women could have implications for understanding and perhaps altering reproductive aging in women.

## Introduction

The hypothalamus is best known as the controller of hormone secretion to regulate essential bodily functions such as reproduction and feeding, and is one of the only brain structures to demonstrate a sexually dimorphic cell population in humans^1^. Recent work in animal models has shown that the hypothalamus also serves as a critical pathophysiologic hub that translates systemic inflammation into neural inflammation, mediating and perpetuating age-related brain and body changes such as cognitive decline and metabolic syndrome^2,3^. Reducing hypothalamic microglial activation by a variety of means including medication, genetic manipulation, and dietary changes, reduces age-related diseases and extends life in animal models, with marked sex differences in this effect^4,5^. To our knowledge, hypothalamic inflammation has not previously been assessed in vivo in humans.

We used Positron Emission Tomography (PET) with 11C-PK11195, a radiotracer sensitive to the translocator protein (TSPO) expressed by activated microglia^6^, to assess the contribution of age and sex to hypothalamic TSPO expression (quantified as non-displaceable binding potential, BPnd) in 43 healthy subjects ranging in age from 23 to 78 (19 female; mean age 55). In addition to chronologic age, we used a machine-learning derived measure of brain biological age (brain age) based on the degree of cortical atrophy present on MRI^7^. We controlled for the potential confound of Body Mass Index (BMI) which differs by age and sex and correlates with whole-brain TSPO expression^8^. We took two imaging approaches: (1) quantification of average hypothalamic BPnd within subjects’ native space using a deep learning technique for accurately segmenting the hypothalamus^9^ and (2) morphing individual brains into template space to localize age and sex effects over the whole brain regardless of traditional neuroanatomic boundaries (Statistical Parametric Mapping; SPM).

**Figure 1:**
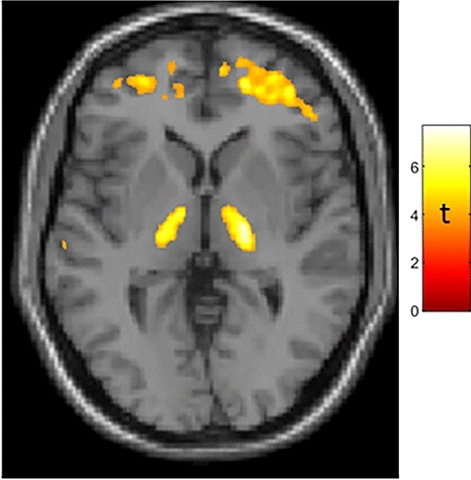
Age-correlated TSPO expression in bilateral thalamus in a mixed-sex group of 43 subjects. Whole-brain statistical parametric analysis with a machine-learning derived measure of brain age^7^ as the regressor of interest, controlling for sex and BMI, shows robust results in bilateral thalamus (peak MNI coordinates: X = 20, Y = − 14, Z = 4 [t = 7.05]; X = 17, Y = − 18, Z = − 4 [t = 6.1]) as well as frontal subcortical regions. T-map is displayed at p_uncorected_ < 0.001. Results were similar when using chronologic age, which was strongly correlated with brain age (r = 0.819, p < 0.001) as the regressor of interest. Additional results of SPM analysis are presented in Supplementary Fig. 1 and Table S2.

In the combined group of women and men (who did not differ in chronologic age, brain age, difference between brain and chronologic age, nor BMI; subject demographics are presented in Supplementary Table 1) SPM analysis showed greater BPnd in association with greater age (both chronologic and brain) in bilateral thalamus (Fig. 1), in accord with prior TSPO PET studies^10–12^. We did not find age-correlated TSPO expression in a probabilistic atlas-defined hypothalamic region of interest^13^ in this mixed-sex group.

**Figure 2:**
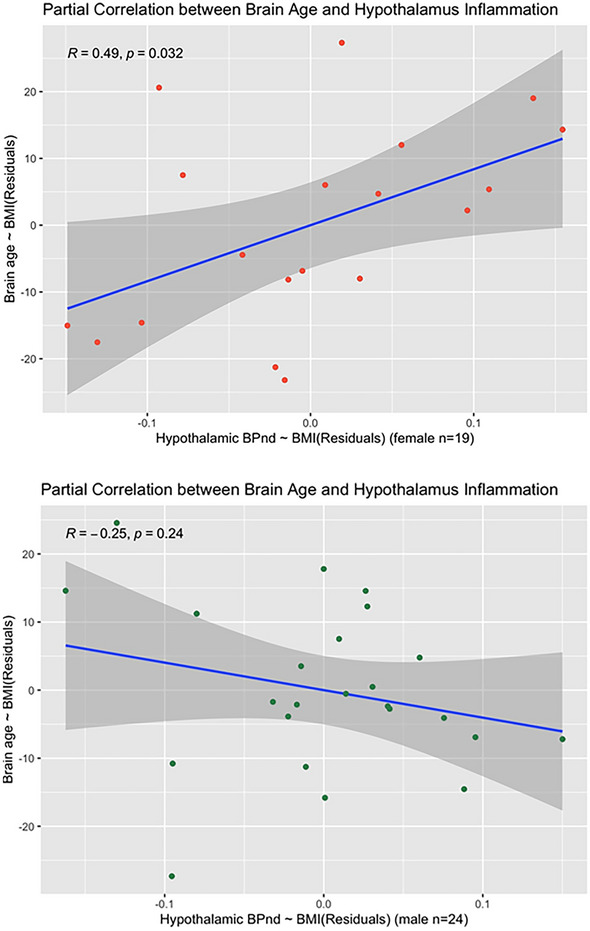
Partial correlation plots showing sex differences in the partial correlation (controlling for BMI) between brain age and hypothalamic TSPO expression, with the hypothalamus segmented in subjects’ native space using a deep learning technique^9^. Top Panel: Women show a significant positive partial correlation between brain age and hypothalamic TSPO expression (r = 0.49; p = 0.03). Bottom Panel: Men show a nonsignificant negative correlation (r = − 0.25, p = 0.24). Fisher Z-comparison showed that these correlations between brain age and hypothalamic TSPO expression different significantly between women and men (p = 0.009).

**Figure 3:**
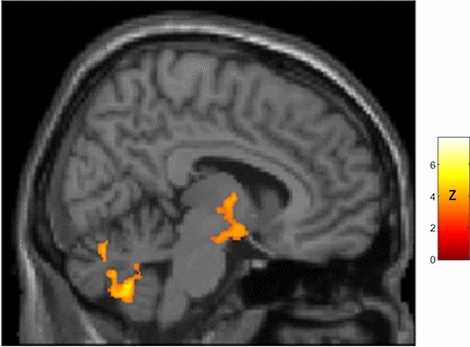
Sagittal view (right of midline; X = 8) of whole-brain statistical z-map showing regions where brain age-correlated TSPO expression was greater in women than men, with prominent results in a region spanning both hypothalamus and thalamus (hypothalamus peak: X = 11, Y = − 7, Z = − 13 [z = 4.2]; thalamus peak: X = 4,Y = − 6, Z = 0 [z = 5.1]; center of gravity in hypothalamus: X = 10, Y = − 5, Z = − 9; additional results of SPM analysis are presented in Supplementary Fig. 2 and Table 5). This sex difference corresponds to significant age-correlated TSPO expression in hypothalamus in women (peak: X = 8, Y = 0, Z = − 12 [t = 3.3; p_corrected_ = 0.002]) but not men. There were no areas of greater age-correlated TSPO expression in men as compared to women.

However, both imaging approaches revealed sex-specific associations of age with TSPO expression in hypothalamus. An optimal regression model including brain age, sex and BMI (R^2^ = 0.457, F(7,35) = 4.203, p = 0.002, AIC = − 223.72) showed that interaction between all of these factors predicted average hypothalamic BPnd (β = − 0.002, p = 0.001). Partial correlation (controlling for BMI) between brain age and hypothalamic BPnd was significant only in women (R = 0.49, p = 0.032), as shown in Fig. 2 A model including chronologic rather than brain age was slightly less accurate (R^2^ = 0.404, F(7,35) = 3.384, p = 0.007, AIC = − 219.72) suggesting greater relevance of biologic than chronologic age to hypothalamic TSPO expression. Additional statistical details are presented in Supplementary Materials. Voxelwise analyses over the whole brain provided convergent results, demonstrating that only women had age-correlated TSPO expression within hypothalamus, and further localizing this sex difference to a region of hypothalamus extending to thalamus, as shown in Fig. 3.

This first assessment of hypothalamic microglial activation in humans demonstrates important age and sex effects.

With respect to age, we found robust age-correlated TSPO expression in thalamus, in accord multiple prior studies^10–12^. This may reflect the thalamus’s role as a relay station for many brain circuits, and the importance of activated microglia in neuroplastic responses to brain and body damage We did not find age-correlated TSPO expression in a probabilistic atlas-defined hypothalamic region of interest^13^ in this mixed-sex group, as might have been expected based on animal studies^2,3^. As discussed below, we detected age-correlated hypothalamic TSPO expression only in women. It is intriguing that in humans, the main focus of age-correlated TSPO expression is thalamus, while in rodents it is hypothalamus^2,3,14^. This difference between rodents and humans, perhaps reflecting evolutionary changes in the size and function of thalamus versus hypothalamus, may be relevant to the appropriateness of using rodents to model human aging and age-related diseases. Rodents are commonly used experimental animal in large part because of their rapid maturity, high reproductive rate, and short lifespan, and these interrelated characteristics (all potentially mediated by hypothalamus) are very different in humans^15^.

We detected age-correlated hypothalamic TSPO expression—stronger for a biologic measure of brain age than for chronologic age—only in women. This novel finding may relate to a stark difference in how women and men age: that women but not men experience loss of fertility—menopause—at mid-life. Menopause is associated with markedly increased risk of cognitive decline and age-related disease including Alzheimer’s^16^ and cardiovascular disease^17^. In rodent models of menopause, estrogen supplementation protects against many of these changes^18,19^. In humans, however, benefits of post-menopausal hormonal replacement therapy remain uncertain despite decades of study^20,21^, again highlighting important differences between humans and rodents, and the need for new paradigms for understanding human reproductive and brain aging. While menopause is generally attributed to changes at the level of the ovary, hypothalamic changes occur early in the menopause transition^22–24^. Our results suggesting a role for hypothalamic inflammation in this change could have implications for understanding and perhaps altering reproductive aging in women.

Our preliminary finding that age-correlated thalamic TSPO expression may extend into hypothalamus in women (Fig. 3) potentially mirrors rodent studies showing hypothalamic inflammation may extend into thalamus^14^. Determining whether inflammation can spread between thalamus and hypothalamus will require longitudinal study and higher resolution techniques.

This study has several significant limitations, including its reliance upon cross section data to study the longitudinal process of aging, lack of information about participants’ menopausal status or reproductive history (e.g. reproductively intact, peri-menopausal, post-menopausal, history of hysterectomy, taking birth control, pregnancy history), and the fact that TSPO is expressed not just by microglia but by several cell types in the brain^25^, and has important functions unrelated to inflammation. In particular, the role of TSPO in steroidogenesis, with estrogen-regulated expression in hypothalamus^26^ may be highly relevant to our findings and to female reproductive aging.

Additional limitations include the use of PET, with its limited spatial resolution, to assess a small structure such as the hypothalamus. This study used a first generation TSPO radiotracer with inferior signal properties to newer tracers^6^. However, TSPO PET is the only available method to assess microglial activation in vivo in humans, and we apply optimal image processing and analysis methods to quantify PET signal^27^ and accurately segment the hypothalamus^9,13^. All statistical analyses assume a linear relationship between age and hypothalamic TSPO expression, which is almost certainly an oversimplification of aging biology^28^.

Current results suggest intriguing differences between rodents and humans, and between women and men, in hypothalamic TSPO expression in aging. Future studies should include longitudinal study design, careful assessment of subject hormonal and reproductive status, and neuroimage acquisition and processing techniques optimized for assessment of small structures such as hypothalamus close to the spatial resolution of PET. Understanding species and sex differences in aging is essential to the development of effective therapies for age-related diseases, and perhaps aging itself.

## Methods

### Subjects

Healthy subjects (n = 43) who underwent 11C-PK11195 Positron Emission Tomography (PK PET) as normal controls for several different studies conducted at Weill Cornell Citigroup Biomedical Imaging Center between 2013 and 2019 (investigating epilepsy, hypertension, chronic fatigue syndrome, Parkinson’s Disease and Gulf War Syndrome) were included in this analysis. All projects complied with ethical standards for human research and were approved by the Weill Cornell Medicine Institutional Review Board (IRB). All subject were free of significant medical, neurologic and psychiatric disease and provided written informed consent prior to participation.

### Image acquisition

PET images were acquired over one hour in list mode, starting at the time of injection of ~ 550 MBq of 11C-PK11195 on a Siemens Biograph PET-CT scanner.

Three-dimensional volumetric T1 weighted BRAVO/MPRAGE MR images (1.2 × 1.2 × 1.2 mm^3^ isotropic voxels) were acquired using a GE Signa or Siemens Skyra 3 T scanner.

### Image processing

PET images were reconstructed into 22 frames and motion corrected using MCFLIRT^29^ within FSL^30^. Smoothing applied to the reconstruction was gaussian FWHM 4 mm. Binding Potential (BPnd) images reflecting the concentration of translocator protein (TSPO) expressed by activated microglia, irrespective of tracer delivery/blood flow, were generated from dynamic PET using a multilinear reference tissue model (MRTM0)^31,32^ implemented in the freely available software package FireVoxel (https://firevoxel.org). The reference tissue time-activity curve was identified via optimized supervised cluster analysis (SVCA)^33^, which is considered the optimal method for analyzing PK PET scans^27^. Each subject’s T1 MRI was linearly co-registered to his/her BPnd image with rigid body transformation in FSL.

### Brain age

An estimate of subjects’ biological (as distinct from chronological) age, termed brain age, was derived from each subject’s MRI using a cloud-based framework based on cortical thickness^7^.

### Whole brain statistical parametric analyses

MRI scans were warped to MNI template space using Advanced Neuroimaging Tools^34^. MRI-derived normalization parameters were applied to co-registered BPnd images to bring them into standard space. Using Statistical Parametric Mapping^35^ (SPM 12), regional BPnd was assessed in the group as a whole and in women and men separately. Within SPM, multiple regression was used to assess brain regions in which BPnd correlated with (chronologic and brain) age. Body Mass Index (BMI) was included as a covariate of no interest because it has been shown to correlate with whole-brain TSPO expression and in association with age and sex^8^. Maps were brain masked to exclude strong extracranial signal which contaminated peripheral cortical regions. SPM analyses were performed without global normalization or grand mean scaling. Results were considered significant at a threshold of p < 0.05 corrected for False Discovery Rate (FDR)^36^ with a minimum cluster volume of 1 cm^3^. Results within the hypothalamic region of a priori interest were considered significant at a small volume corrected threshold of p < 0.05. The hypothalamic region of interest (ROI) was defined based on a high resolution probabilistic atlas of bilateral hypothalamus^13^ threshold at 50%. T-maps generated using SPM were converted to Z-maps within SPM to directly compare age-correlated TSPO expression between women and men. Results were considered significant at Z > 3. XJview (https://www.alivelearn.net/xjview) was used to calculate the center of gravity of clusters and generate figures.

### Native space analyses

Regional BPnd was quantified within hypothalamus within the subject’s native space using Freesurfer^37^ with a highly-accurate deep learning technique for segmenting the hypothalamus^9^. Segmented regions were transformed to PET space with the inverse transformation matrix from the co-registration step and eroded 1 mm in plane to minimize partial volume effects.

### Statistical analyses

Analyses were performed in R. All results were considered significant at p < 0.05.

T-tests were used to assess differences between men and women in age, brain age, difference between chronologic age and brain age, and BMI.

Stepwise linear regression models were built to assess if sex, age (chronologic and brain), BMI and their interactions significantly predicted average hypothalamic BPnd (from the native space analysis). These predictors were chosen based on prior evidence implicating them in hypothalamic structure/function and whole brain TSPO PET^8^. The optimal regression model was chosen based on Akaike Information Criterion (AIC). To further assess significant effects, partial correlations between hypothalamic BPnd and age, controlling for BMI, were assessed separately in women and men using Pearson’s test, and compared using Fisher Z-comparison.

## Supplementary Information

Supplementary Information.

## Data Availability

All data, in de-identified format, is available upon written or emailed request to the corresponding author.
