# Supplementary material for: Positron Emission Tomography reveals age-associated hypothalamic microglial activation in women
Source: Sci Rep. 2022 Aug 3;12:13351. doi: 10.1038/s41598-022-17315-8 (PMC9349172; doi:10.1038/s41598-022-17315-8)
Supplement: Supplementary file 1 — Supplementary Information. [file 41598_2022_17315_MOESM1_ESM.docx]

**Positron Emission Tomography reveals age-associated hypothalamic microglial activation in women.**

**Supplementary Results**

| **Supplementary Table 1.** Subject demographics. | | | |
| --- | --- | --- | --- |
|  | **Women (n=19)** | **Men (n=24)** | **T value; p value** |
| **age** | 56.32 | 54.01 | 0.50; 0.66(NS) |
| **Brain age** | 50.98 | 46.79 | 0.97; 0.34(NS) |
| **Difference between brain and chronologic age*** | -5.33 | -7.21 | -0.64; 0.52(NS) |
| **BMI** | 25.75 | 27.584 | -1.39; 0.17(NS) |
| *Brain age is younger than chronologic age. This is expected in subjects selected to serve as controls because they are healthy. | | | |

| **Supplementary Figure 1.** Sectional view of whole-brain statistical t-map showing brain regions where 11C-PK11195 BPnd correlated positively with brain age in 43 healthy subjects. Sex and BMI were included as nuisance covariates. Map is displayed at a threshold of p_uncorrected_<.001 and minimum 1cm^3^ cluster volume. The axial image, highlighting results in bilateral thalamus (clusters #1 and #6 in supplemental Table 4) is also presented as Figure 1 in the main manuscript. Coronal and sagittal views show the full extent of this cluster as well as results in bilateral frontal and temporal subcortical regions. | |
| --- | --- |
| 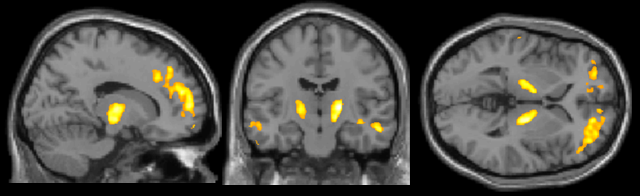 | 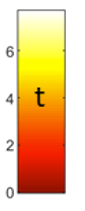 |

| **Supplementary Table 2.** Brain regions where 11C-PK11195 BPnd correlated positively with brain age in 43 healthy subjects at a threshold of p<.05 corrected for False Discovery Rate (FDR) over the whole brain and cluster volume > 1cm^3^. Sex and BMI were included as nuisance covariates. Results in bilateral thalamus (clusters #1 and #6) are visible in Figure 1 in the main manuscript and in full sectional view in Supplementary Figure 1. Results were similar when using chronologic rather than brain age as the regressor of interest. | | | | |
| --- | --- | --- | --- | --- |
| **cluster** | **# voxels** | **Peak t** | **Peak MNI coordinates** | **Region** |
| **1** | **2740** | **6.97** | **20 -14 4** | **R thalamus** |
| 2 | 119140 | 6.6 | 13 52 28 | R frontal subcortical |
| 3 | 6482 | 5.81 | -10 52 -12 | L frontal subcortical |
| 4 | 3318 | 5.71 | -56 -36 -6 | L temporal subcortical |
| 5 | 1180 | 5.69 | 50 18 18 | R frontal subcortical |
| **6** | **1757** | **5.42** | **-14 -11 4** | **L thalamus** |
| 7 | 2651 | 5.33 | 55 15 16 | R temporal subcortical |

| **Supplementary Table 3:** Additional statistical results relating Brain Age to Hypothalamic TSPO expression. **A:** Full results for optimal regression model using brain age, sex, BMI and their interactions to predict hypothalamic 11C-PK11195 BPnd. **B:** Uncorrected correlation between brain age and hypothalamic 11C-PK11195 BPnd in women and men. | |
| --- | --- |
| A. | 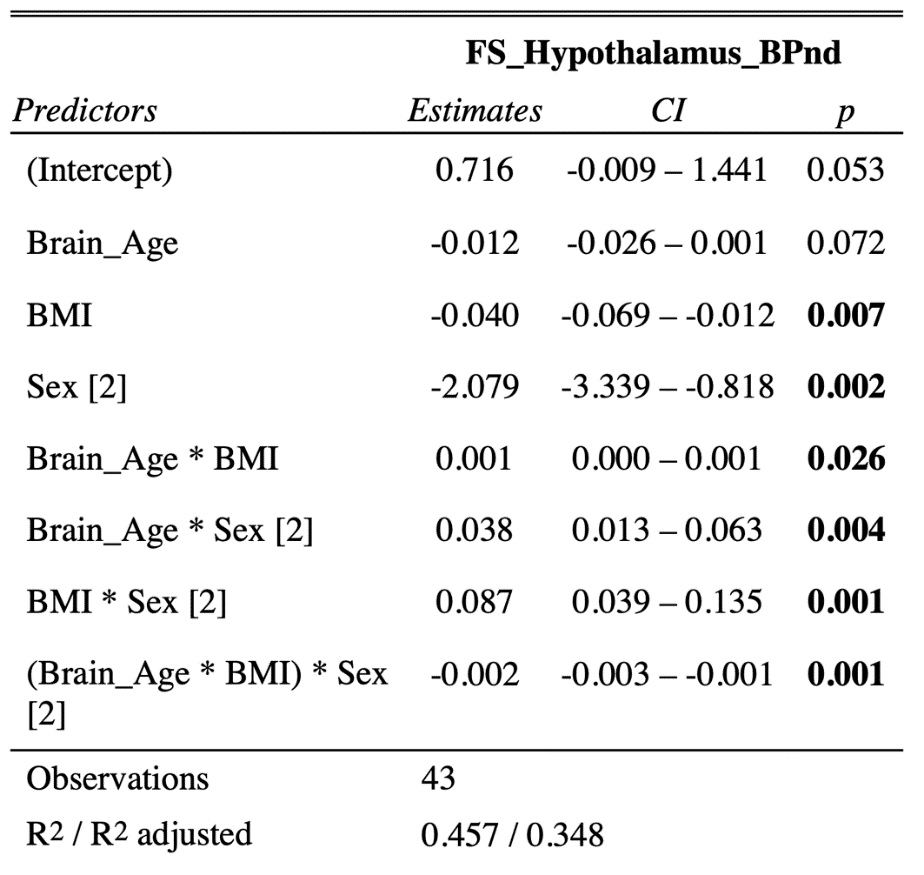 |
| B. | Pearson correlation (uncorrected for BMI) between brain age and hypothalamic TSPO: female (r = 0.36, p = 0.13); male (r = -0.23, p = 0.27). |

| **Supplementary Table 4.** Additional statistical results relating Chronologic Age to Hypothalamic TSPO expression. **A:** Full results for optimal regression model using chronologic age, sex, BMI and their interactions to predict hypothalamic 11C-PK11195 BPnd. **B:** Uncorrected correlation between chronologic age and hypothalamic 11C-PK11195 BPnd in women and men. | |
| --- | --- |
| A. | 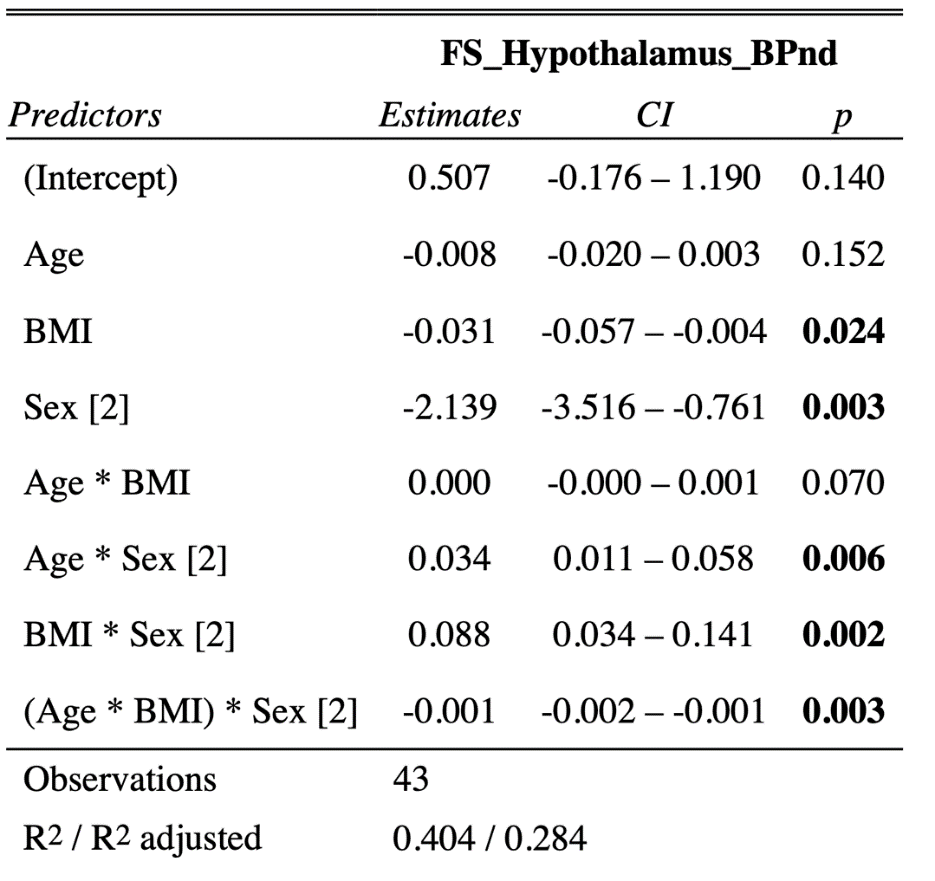 |
| B. | Pearson correlation (uncorrected for BMI) between chronological age and hypothalamic TSPO: female (r = 0.37, p = 0.12); male (r = -0.14, p = 0.52) |

| **Supplementary Figure 2.** Sectional view of whole-brain statistical z-map showing regions where brain age-correlated 11C-PK11195 BPnd was greater in women (n=19) than men (n=24). The sagittal view, highlighting results in the R>L hypothalamic-thalamic region (cluster #4 in supplemental Table 4), is also presented as Figure 3 in the main manuscript. Coronal and axial views show the full extent of this cluster as well other regions (left caudate, several right cortical and subcortical regions) outside the hypothalamic region of interest. Map is displayed at a threshold of z=3 and minimum 1cm^3^ cluster volume. | | | | | |  |
| --- | --- | --- | --- | --- | --- | --- |
| 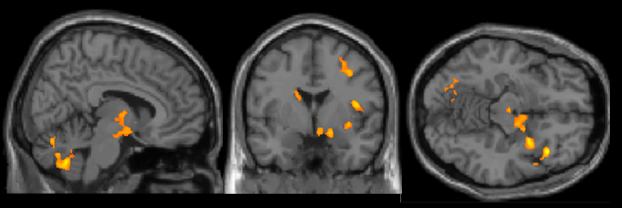 | | | | | 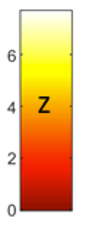 |  |
| **Supplementary Table 5.** Brain regions where brain age-correlated 11C-PK11195 BPnd was greater in women (n=19) than men (n=24) at a threshold of z=3 and minimum 1cm^3^ cluster volume. This Z map was generated by converting single sex t-maps (brain age as regressor of interest; nuisance covariate = BMI) to Z maps and then subtracting them. There were no regions of greater brain age-correlated TSPO expression in men as compared to women. Cluster #4 includes the hypothalamic region of interest, as shown in sagittal view as Figure 3 in the main manuscript and in full sectional view in Supplementary Figure 2. Results were similar when using chronologic rather than brain age as the regressor of interest. | | | | | | |
| **cluster** | **# voxels** | **Peak z score** | **Peak MNI coordinates** | **Region** | | |
| 1 | 4180 | 5.458 | 5 -67 -42 | R cerebellum; may also reflect TSPO expression in walls of superior sagittal sinus. | | |
| 2 | 3077 | 6.1993 | -30 -68 -15 | L cerebellum; may also reflect TSPO expression in walls of superior sagittal sinus. | | |
| 3 | 4985 | 5.5828 | 43 -3 13 | R insula / frontal subcortical | | |
| **4** | **3548** | **5.4865** | **31 5 -10** | **R > L subcortical including thalamus and hypothalamus and extending to R medial temporal. Center of gravity is in hypothalamus (15 -3 -9)** | | |
| 5 | 1346 | 4.8092 | 18 -73 2 | R occipital / lingual | | |
| 6 | 1308 | 4.6901 | -4 41 16 | Anterior Cingulate | | |
| 7 | 1527 | 4.5926 | 51 -57 5 | R temporal-parietal subcortical | | |
| 8 | 1403 | 4.7619 | -12 5 14 | L caudate | | |
| 9 | 1424 | 5.9667 | 53 -40 27 | L parietal / supramarginal | | |
| 10 | 1723 | 7.6688 | 28 34 42 | R middle frontal | | |
| 11 | 1265 | 5.3414 | 34 -9 46 | R frontal subcortical | | |
